# Ignition and Combustion Characteristic of B·Mg Alloy Powders

**DOI:** 10.3390/ma15082717

**Published:** 2022-04-07

**Authors:** Yusong Ma, Kaichuang Zhang, Shizhou Ma, Jinyan He, Xiqiang Gai, Xinggao Zhang

**Affiliations:** State Key Laboratory of NBC Protection for Civilian, Beijing 102205, China; mayusong111@163.com (Y.M.); 18333109527@163.com (K.Z.); 13910011793@139.com (S.M.); hejinyanfhy@163.com (J.H.); gaixq@126.com (X.G.)

**Keywords:** boride, MgB_2_, mechanical alloying, ignition, combustion, flame temperature

## Abstract

Boron and its alloys have been explored a lot and it is expected that they can replace pure aluminum powder in the energetic formulation of active materials. MgB_2_ compounds were prepared and characterized by a combination of mechanical alloying and heat treatment. The ignition and combustion of boron–magnesium alloys were studied with the ignition wire method and laser ignition infrared temperature measurement. The results show that MgB_2_ has good ignition characteristics with maximum ignition temperatures obtained by the two various methods of 1292 K and 1293 K, respectively. Compared with boron, the ignition temperature of MgB_2_ is greatly reduced after alloying. The ignition reaction of MgB_2_ mainly occurs on the surface and the ignition process has two stages. In the initial stage of ignition, the large flame morphology and combustion state are close to the combustion with gaseous Mg, whereas the subsequent combustion process is close to the combustion process of B. Compared with boron, the ignition temperature of MgB_2_ is greatly reduced which suggests that MgB_2_ may be used in gunpowder, propellant, explosives, and pyrotechnics due to its improved ignition performance.

## 1. Introduction

As an energetic material, combustible metal powder is widely used in gunpowder, propellant, explosives, and pyrotechnics [1,2,3,4,5,6]. The reaction of metal powder and oxidant can provide the power for gun projectile launching and rocket missile propulsion. It is the power energy of the warhead explosion and the energy source that realizes smoke interference and combustion damage. The performance of metal powder to a large extent determines the level of combat technology of weapons and equipment, and is the core technology to achieve long-range strike and high-efficiency damage, and is the key material basis for the development of advanced weapons and equipment.

Among the combustible metal powders, magnesium is easy to ignite and burn but has a low calorific value. Compared with magnesium, boron has a very high calorific value. Its mass calorific value is 2.3 times that of magnesium and 1.9 times that of aluminum, and its volume calorific value is 3.09 times that of magnesium and 1.66 times that of aluminum. However, the ignition temperature of boron is high. In the combustion process, the liquid boron oxide that is generated hinders the reaction between boron and the oxidizer; moreover, the boron particles are easy to agglomerate, which makes the combustion difficult to continue. These factors lead to the low combustion efficiency of boron and underutilization of boron’s high energy [3,7,8]. To solve the above problems, we prepared a new alloy material based on the synergistic effect with lower ignition temperature, faster combustion speed, weaker combustion agglomeration, and more complete combustion than a single metal powder material [9,10,11,12], important for improving the performance of propellants, explosives, and pyrotechnics.

The preparation of boron-containing alloys, such as Al–Ti–B and Ti–B alloys, has been reported by previous studies [13,14,15,16,17]. Shtessel et al. [10] prepared a series of Al–Mg, Al–Mg–H, and Al–B alloys by high-energy ball milling. Birol et al. [18] also prepared Al–B alloys from Al and B_2_O_3_ by ball milling, whereas Korchagin et al. [19] synthesized Ni_3_B using high-energy ball milling of a mixture of Ni and B powders. Guo et al. prepared MgB_2_ by a high-temperature sintering method at 1173 K [16,17].

Many methods based on differential thermal analysis (DTA) have been reported for estimating the ignition temperature of materials. In the mature and stable method, the ignition temperature is obtained by extrapolating the reaction temperature. However, this approach is subject to atmospheric factors, heating rate, and other parameters during testing. Shoshin et al. [20] studied the ignition of Al–Ti alloy in the air by coating a wire with the powder and measuring its temperature during electrical heating. Other researchers used lasers of different powers to measure the ignition and combustion characteristics [21,22,23]. Zhao [24] used a contact thermocouple to assess the ignition temperature of metal fuel in different atmospheres. Yang and co-workers [25] used planar flame burners to ignite modified boron and examine its combustion characteristics. Whittaker et al. [13] found that AlB_2_ is an energetic fuel with a high heat of combustion. Arkhipov et al. [26] studied the ignition and combustion of propellants of Al, B, and Al–B powders with binders with oxidizing agents (ammonium perchlorate or ammonium nitrate).

In this study, MgB_2_ was prepared by a combination of mechanical alloying and heat treatment, and the ignition and combustion of boron–magnesium alloys were studied with the ignition wire method and laser ignition infrared temperature measurement.

## 2. Materials and Methods

### 2.1. Materials and Instruments

Atomized spherical magnesium powder (Mg) has a particle size <45 μm and purity of 99.9%, Shanghai Shuitian Material Technology Co., Ltd., Shanghai, China. The amorphous boron powder (B) had a particle size of 1~3 μm and purity of 99.7%, Liaoning Yingkou Fine Chemical Company. MgB_2_ was prepared by a combination of mechanical alloying and heat treatment.

The following instruments were used: a vibrating high-energy ball mill (Beijing Nonferrous Metals Research Institute, Beijing, China), a vacuum operation box (ZKX-2, Nanjing Nanda Instrument Factory, Nanjing, China), a tube furnace (SGL-1700-II, Shanghai Jujing Precision Instrument Manufacturing Co., Ltd., Shanghai, China), an X-ray diffractometer (D8 ADVANCE, Bruker, Karlsruhe, Germany), an S-4800 cold field emission scanning electron microscope (Hitachi, Tokyo, Japan), and a microcomputer automatic calorimeter (TRHW-7000E, Hebi City Tianrun Electronic Technology Co., Ltd., Hebi, China).

### 2.2. Preparation of MgB_2_

The Mg and B powders in a mole ratio of 1:2 were placed in a stainless steel ball mill jar. The ball mill tank was sealed with an O-ring and passed through a flow of 0.1 MPa argon three times for three minutes. The vibrating high-energy ball mill used stainless steel balls of either φ10 mm or mixed sizes (φ2 mm, φ5 mm, and φ10 mm in a mass ratio of 1:1:3) at 20:1 mass to the powder materials. Powder loading and sampling operation were both carried out in a vacuum operation box filled with argon gas. During milling, the ball mill had a three-dimensional motion of rotational oscillation and vibration. The ball milling time was 12 min, and after milling, the powder was sealed in a vacuumed quartz glass tube and placed in a tube furnace with a flowing Ar atmosphere at 20 mL/min. The furnace was heated from room temperature (298 K) to 853 K at a rate of 5 K/min and held for 10 h.

### 2.3. Performance Test

The structure of the product was analyzed by X-ray diffraction (XRD) using Cu target Kα radiation (0.15406 nm), a working voltage of 40 kV, a working current of 20 mA, and 2θ angle scan range of 10–90° in steps of 0.02° at a scanning speed of 0.5°/min.

**Figure 1:**
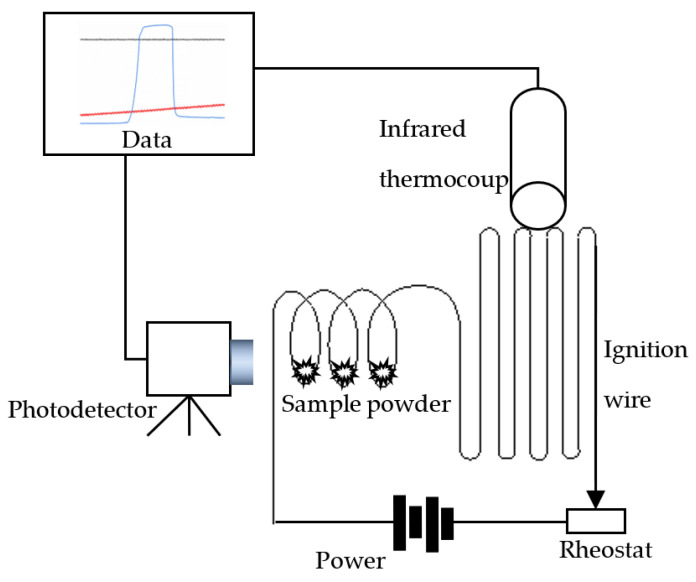
The sketch of the ignition temperature tester.

The setup for measuring the ignition temperature is shown in Figure 1. The sample powder was placed on the wound ignition wire. After the power was turned on, the ignition wire was gradually heated, and we assume that at the point of powder ignition the powder and the wire had the same temperature. The timing of the ignition was monitored by a photodetector, and the temperature change in the ignition wire was measured by an infrared thermocouple. To avoid the heat released by sample combustion interfering with the temperature measurement, the infrared thermocouple monitored other parts of the ignition wire not covered with the sample powder. The light intensity and temperature data during the experiments were recorded by the data acquisition instrument.

The ignition wire was a 0Cr27Al7Mo2 iron chrome aluminum alloy wire 0.5 mm in diameter. An SYS480S36 DC switching power supply with a maximum output of 480 W directly heated the ignition wire. The supply had overcurrent protection, and its current was not less than 10 A to provide instantaneous large current through the ignition wire. To facilitate the determination of the ignition starting time, the DC power supply was controlled by a solid-state relay (SSR200D40, Hangzhou Guojing Technology, Hangzhou, China). The temperature was measured using an infrared thermocouple (OS37-20-K, OMEGA Company, Austin, TX, USA) supplemented by a temperature transmitter (TXDIN1620, OMEGA Company, Austin, TX, USA), control line, and switching power supply (YMGUD-2030LIXA, Yongming Power Company, Taiyuan, China). The infrared thermocouple output was the K-type, the temperature range was 533–1923 K, and the temperature accuracy was ±2% in the linear zone and ±5% in the other ranges. The spectra of combustion for Mg, B, and MgB_2_ powders had a wavelength range of 300–900, 450–650, and 300–800, respectively, with peak wavelengths of 497.7, 546, and 546.8/579 nm. The test equipment used Thorlabs’ DET10A photodiode, which is a silicon-based photodetector with a detection wavelength covering the visible range and a peak response at 730 nm in the near-infrared region. The data acquisition was carried out with a NI9222 card (National Instruments, Austin, TX, USA) with a maximum sampling rate of 500 K, and LABVIEW software.

**Figure 2:**
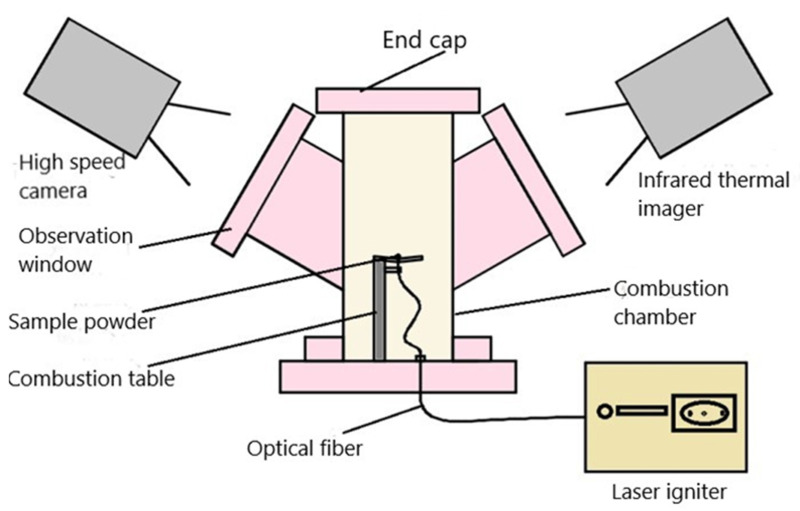
Schematic diagram of laser ignition experimental system.

The laser ignition system consisted of three parts: laser igniter, combustion chamber, and data acquisition device, as shown in Figure 2. The laser igniter included three main parts: fiber-coupled semiconductor laser, laser power supply, and an optical coupler. The microscopic high-speed photography-infrared thermography synchronization device recorded the flame, the sample morphology, and the temperature field during the MgB_2_ ignition and combustion processes. The thermal imaging camera (SC325, FLIR Systems, Inc., Tigard, OR, USA) could record in a temperature range up to 2273 K. The high-speed camera (FASTCAM-APX-RS, Photron, Arizona, CA, USA) had an AF Micro-Nikkor 60 mm f/2.8 D macro lens. To facilitate the observation of the ignition and combustion process of small particles, an AF Micro-Nikkor 60 mm f/2.8 D lens with macro function (Nikon Corporation, Tokyo, Japan) was used. In the experiment, a continuous laser with a power of 4 W was used to ignite and burn 50 mg of micro-clustered sample in an air atmosphere of 0.1 MPa. Infrared thermal imagers and high-speed cameras simultaneously detected changes in the flame topography and temperature field. The surface morphology of the particles was directly observed by high-speed photography plus the macro lens, and the shooting speed was 2000 frames per second.

The micro-machine automatic calorimeter was used in an oxygen atmosphere of 3 MPa. The volume of the oxygen bomb was 300 mL, and the sample amount of the single test was 0.5 g. The combustion heat tests were performed on the samples of Mg, B, and MgB_2_.

## 3. Results

### 3.1. Preparation and Characterization of MgB_2_

**Figure 3:**
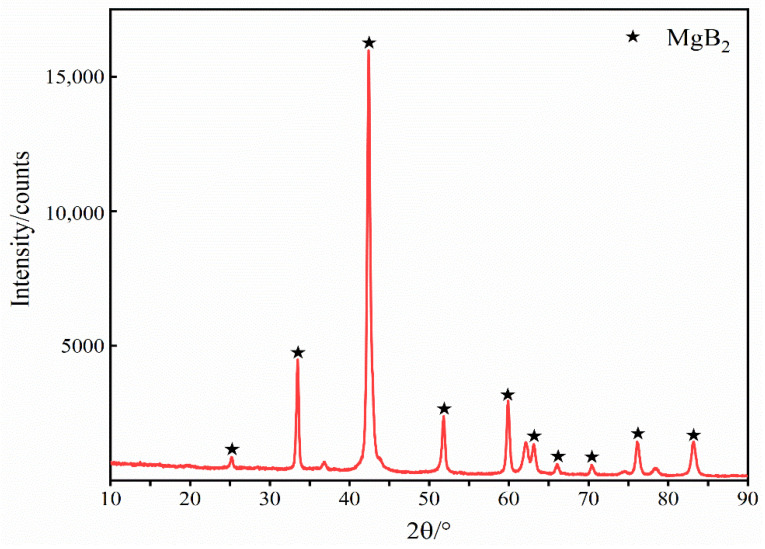
Comparison of the XRD patterns of prepared MgB_2_ and PDF Card.

The XRD spectrum of the prepared MgB_2_ was compared with the PDF card standard spectrum by XRD spectrum software Jade, as shown in Figure 3.

The position of the spectrum peak and the corresponding intensity are consistent with the standard spectrum peak in the PDF card, and there is basically no spurious peak, indicating that the product is MgB_2_ with high purity.

### 3.2. Experimental Study of MgB_2_ Ignition Performance

**Figure 4:**
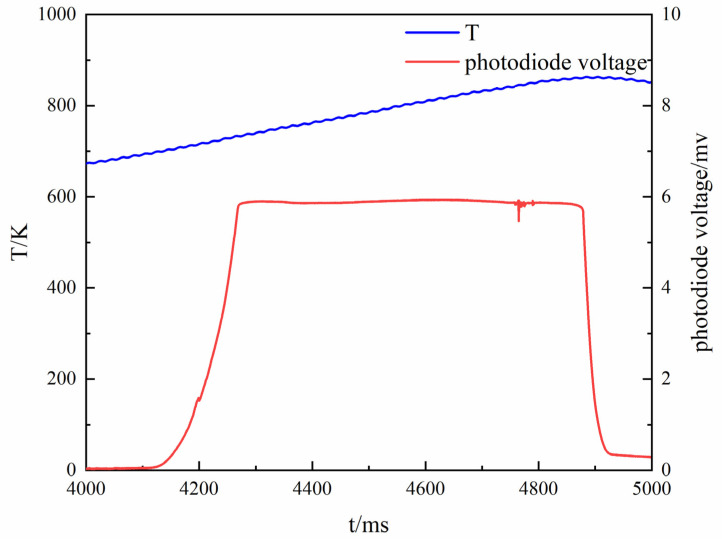
The ignition temperature curves of Mg powder.

In this paper, the test device was designed to completely close the switch, and the resistance wire started to heat up as the starting moment; that is, the solid-state relay was fully turned on, and the control voltage continued to be higher than 3.5 V as the heating start time. Theoretically, the temperature and intensity of the powder change during the powder ignition process, and the temperature at the moment of the mutation is the ignition temperature. Since the temperature of the heating wire is increased after the electric wire is energized, it gradually becomes brighter. It is known that the output voltage of the photodiode is not more than 0.3 V during the heating process of the ignition wire. Therefore, the design of the test device is such that the light intensity continuously exceeds 0.3 V as the ignition starting time. The first peak of the light intensity is the moment when the powder is completely ignited, and the average temperature of the corresponding ignition wire is the powder ignition temperature. Since the infrared thermocouple test delay is 80 ms, the temperature curve is shifted back from the start time by 80 ms during data processing. The period of temperature change of the ignition wire is about 20 ms; that is, the infrared thermocouple outputs effective temperature measurement data after continuous testing for 20 ms. If the powder ignition time is less than 20 ms after ignition, start time is taken as the ignition duration, and the corresponding ignition wire temperature is the ignition temperature. The obtained magnesium powder ignition temperature curve is shown in Figure 4.

The blue curve is the temperature, and the red curve is the voltage value of the photodiode. It can be seen from the test that the average ignition temperature of magnesium powder is 719 K, which is the same value reported in the literature [25]. Therefore, the feasibility of the test device is verified by the ignition temperature test of magnesium powder.

**Figure 5:**
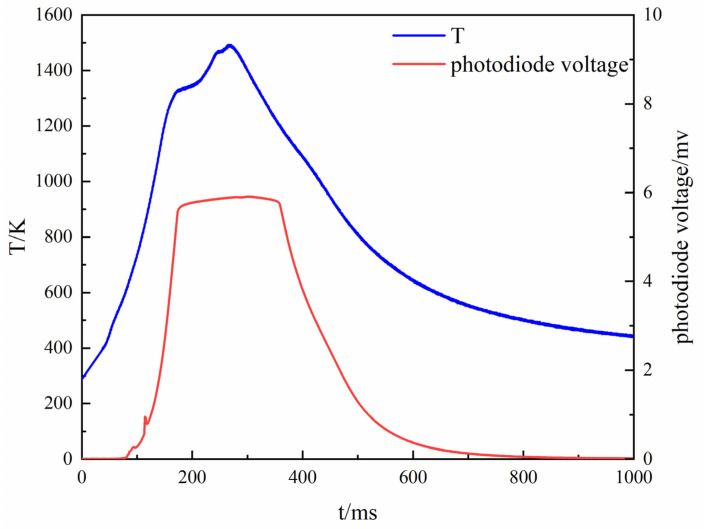
Curves of MgB_2_ ignition temperature test.

The procedure of the MgB_2_ ignition temperature test included adjusting the varistor shown in Figure 1 and reducing the loop load resistance by 1 Ω. The obtained curve shows that the ignition temperature of the boron-magnesium alloy is 1292 K, as shown in Figure 5.

**Figure 6:**
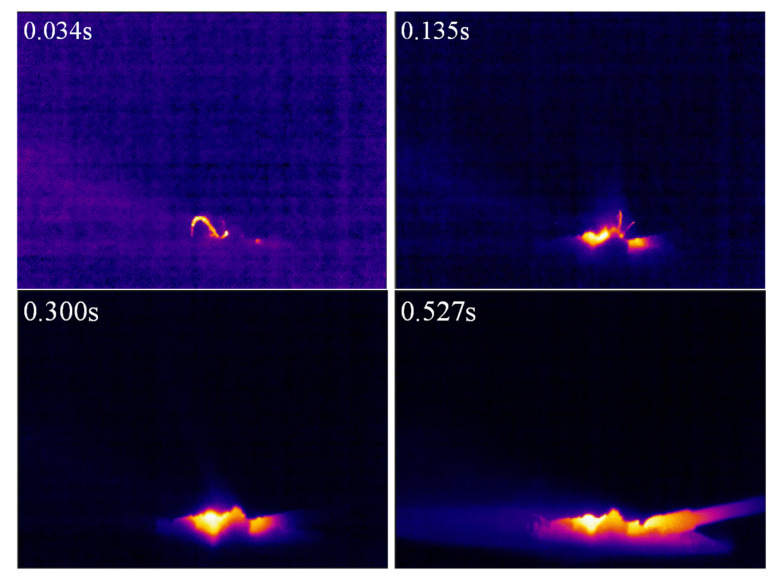
Infrared temperature measurements during ignition and combustion of the of the MgB_2_ sample.

**Figure 7:**
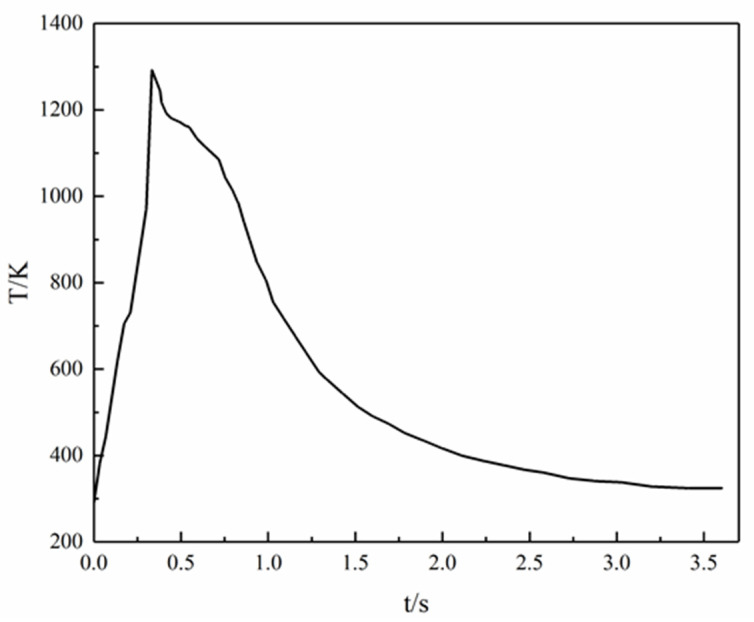
The curve of MgB_2_ sample ignition temperature.

In the laser ignition experimental system shown in Figure 2, the micro-cluster of the MgB_2_ sample was placed on the combustion table. The laser fiber was adjusted to face the center of the micro-cluster, and then the focal length of the macro lens was adjusted to ensure that the micro-cluster was in the focus position, while simultaneously turning on the infrared thermal imager. The ignition experiment started by triggering the high-speed camera and turning on the laser power. The temperature during ignition and combustion of the MgB_2_ sample in the infrared thermal imaging test is shown in Figure 6 and the infrared temperature measurements at different time points are shown in Table 1 and Figure 7.

The MgB_2_ sample reached the highest ignition temperature of 1293 K at 0.333 s, and a flame appeared on the surface of the sample. Macek et al. studied the ignition and combustion of boron particles which showed that the ignition temperature of boron is about 2000 K under the air atmosphere [27]. The boron powder is surrounded by the highly viscous boron oxide B_2_O_3_ produced during the ignition and combustion process, so that the ignition and combustion of boron powders are difficult to carry out. Compared with boron, the MgB_2_ sample ignition temperature was lower by 700 K, indicating that the ignition performance is significantly improved after boron alloying.

### 3.3. Study of the Combustion Properties of MgB_2_

**Figure 8:**
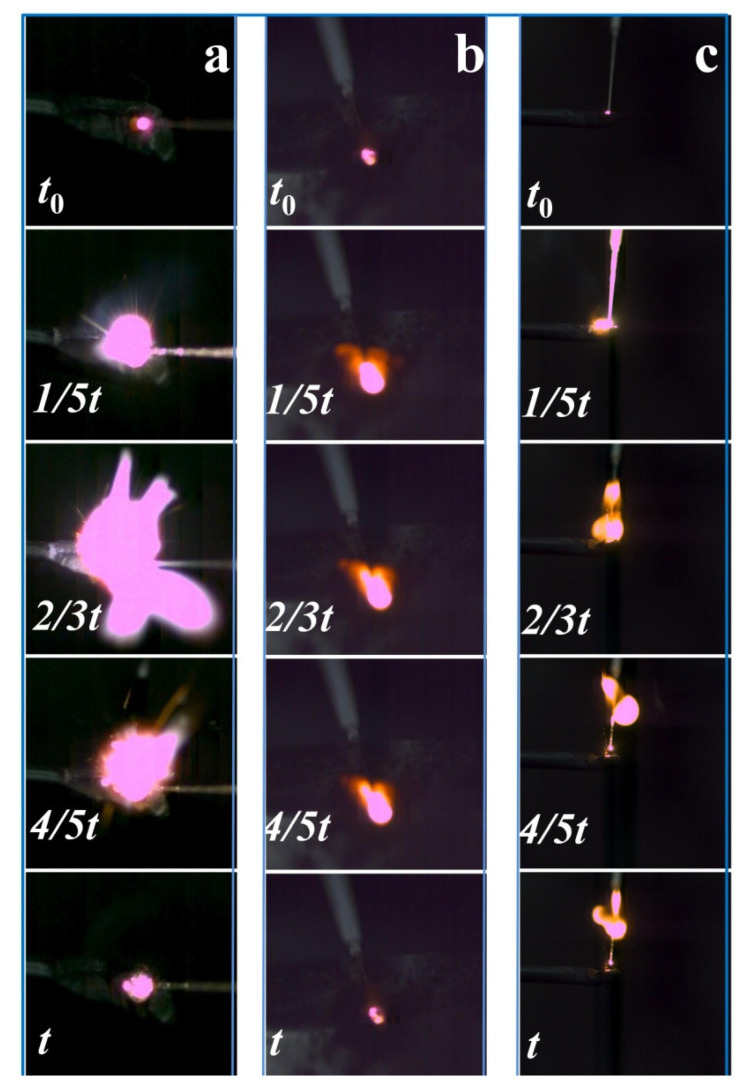
Burning flame morphology of Mg (**a**), B (**b**), and MgB_2_ (**c**) samples.

Infrared thermal imagers and high-speed cameras were used to simultaneously detect changes in flame topography and temperature field distribution. Among them, the initial ignition timing of the sample is t_0_, and the continuous burning time of the sample is t. A comparison of the high-speed photographic test results of the ignition reactions of Mg, B, and MgB_2_ samples is shown in Figure 8.

It can be seen from Figure 8 that the flame size of Mg is large, and the flame size of B is small during the combustion process, and the flame morphology does not change much during the combustion process, whereas the flame of the MgB_2_ sample is large at the initial stage. The flame of the MgB_2_ sample becomes smaller after 2/3 t, but the duration is still longer. This is because Mg has a low melting point and a low boiling point, and is easily melted and vaporized during ignition and combustion, so that gaseous Mg is easily formed during combustion. Boron has a relatively high melting point and boiling point, and is not easily vaporized during ignition and combustion, and its combustion process is close to solid phase combustion. The combustion of the MgB_2_ sample can be seen as a two-stage process. During the ignition and initial combustion stages, the MgB_2_ sample can form part of the gaseous Mg, thus forming a larger flame, and then approaching the solid phase combustion with B.

The ignition experiments of the Mg, B, and MgB_2_ samples were repeated five times, combined with the infrared test data, and the characteristic parameters thereof are shown in Table 2. Mg burns fastest whereas B burns the slowest. MgB_2_ sample burns at a medium speed, but its maximum combustion temperature is close to Mg.

## 4. Conclusions

An ignition study of the boron–magnesium alloy was carried out by using the ignition wire method and the laser ignition infrared temperature measurement method. Compared with boron, the ignition temperature of MgB_2_ is greatly reduced, suggesting improved ignition performance. The main conclusions obtained can be summarized as:

1. The results show the maximum ignition temperatures of MgB_2_ obtained by the two methods are 1292 K and 1293 K, respectively.
2. The calculation results of temperature change during MgB_2_ laser ignition were in good agreement with the experimental results, indicating that the calculation model is capable of describing the temperature change of the particle ignition process.
3. The flame morphology and combustion state are close to the combustion with gaseous Mg in the initial stage. However, the subsequent combustion process is close to the combustion process of B. Compared with Mg and B, the MgB_2_ sample burns at a medium speed.

## Figures and Tables

**Table 1:** Infrared temperature of the MgB_2_ sample at different time points.

t(s)	0	0.034	0.068	0.135	0.174	0.209	0.300	0.333	0.527
T(K)	290	381	445	617	705	730	969	1293	1163

**Table 2:** Characteristic ignition parameters of Mg, B, and MgB_2_ samples.

	Sample	Mg	B	MgB_2_
Parameter				
Burning time(s)		0.41	0.86	0.53
Ignition temperature(K)		719	2000	1293

## Data Availability

The data presented in this study is available on request from the corresponding author.
